# Abdominal obesity, serum estradiol and cardiovascular risk among Nigerian postmenopausal women: a cross-sectional study

**DOI:** 10.4314/ahs.v23i3.12

**Published:** 2023-09

**Authors:** Omolara T Lewechi-Uke, Ikeoluwapo O Ajayi, Joshua O Akinyemi

**Affiliations:** 1 Department of Family Medicine, University College Hospital, Ibadan; 2 Department of Epidemiology and Medical Statistics, Faculty of Public Health, University of Ibadan, Nigeria

**Keywords:** Cardiovascular risk, postmenopausal women, abdominal obesity

## Abstract

**Background:**

Rates of cardiovascular (CV) disease mortality is usually higher in men but this equalizes with that of women following menopause.

**Objectives:**

This was to determine the contribution of abdominal obesity and estradiol to cardiovascular risk in postmenopausal women (PMW) as well as estimate their CV risk profile.

**Methods:**

271 consenting PMW were recruited consecutively into this cross-sectional hospital-based study. Data relating to their socio-demography, blood pressure and anthropometry was obtained and laboratory analysis of lipid profile and serum estradiol was done. Cardiovascular risk of participants was estimated using standardized calculators.

**Results:**

Mean age of participants was 57.8±5.5 years. Significant correlation existed between each of triglyceride (Positive), High Density Lipoprotein (negative) and Waist-Hip-Ratio (WHR) (p=0.001 and 0.000 respectively). Hypertension and dyslipidaemia were significantly associated with WHR (p=0.01 and 0.031 respectively). Significant negative correlation existed between CV risk profile and serum estradiol (rs = -0.140, p = 0.028). Ten-unit increase in WHR was associated with two-fold risk of hypertension (OR> 1.73, C.I.= 1.13-2.66). A unit change in age was associated with 0.61 increase in TC.

**Conclusion:**

Abdominal obesity and serum estradiol significantly influence cardio-metabolic risk. Newer risk calculator which incorporates factors peculiar to women such as serum E2 is hereby recommended.

## Introduction

Cardiovascular diseases (CVDs) form a significant proportion of non-communicable diseases. Rates of CVD mortality increase sharply with age in both men and women, with higher rates in men than women before menopause however this gender specific difference in mortality narrows significantly after menopause^1^. Cardiovascular diseases have long latency period and are usually precipitated by risk factors. Cardiovascular (CV) risk refers to the likelihood of occurrence of an unwanted CV event such as stroke or myocardial infarction.

Various markers of cardiovascular risk have been identified which include novel markers (C-reactive protein, Growth Differentiation Factor, GDF-15 and Brain Natriuretic Peptide, BNP) and the traditional markers (smoking status, hypertension, diabetes mellitus and raised total cholesterol). Obesity, specifically truncal obesity (also known as abdominal obesity) is an important determinant of CV risk, evidenced by its inclusion as a parameter in the cardiometabolic syndrome. Abdominal obesity occurs due to accumulation of visceral adipose tissue (VAT) which is a hormonally active component of total body fat. Abdominal obesity carries greater risk of future CVD events and diabetes mellitus than peripheral or gluteofemoral obesity^2^. Naturally, the post-menopausal status is associated with a higher tendency to gain weight with resultant redistribution of fat cells which leads to increased lying down of visceral fat. 3. This suggests that abdominal obesity could be an important predictor of CV risk in postmenopausal women (PMW). Other CVD risk marker specific to women include history of pregnancy complications (preeclampsia, eclampsia, gestational DM or PIH), use of oral contraceptives or postmenopausal hormonal replacement therapy. The fore-going suggests the possibility of using serum E2 level as a laboratory determinant of CV risk profile in women (especially in PMW).

Cardiovascular risk profile assessment refers to the use of clinical and laboratory parameters to predict the likelihood of developing a cardiovascular event at a particular time in the future, with the goal of preventing or delaying such occurrence by treating or modifying risk factors 4,5. These risk factors can be used singly or together in known CV risk calculators.

Sub-Saharan Africa (SSA) is not left out in the worldwide rise in CVD. More than 85% of deaths due to NCDs occur in SSA where poverty and ignorance, with its associated health consequences such as HIV/AIDS, malaria and Tuberculosis already pose a great challenge 6. There is hence, the need to identify and make use of simple available clinical and laboratory predictor of CV risk as means of early screening for CVD with a view of early intervention.

This study seeks to determine the contribution of abdominal obesity and estradiol to cardiovascular risk in participants as well as estimate their CV risk profile (using established tools) and elucidate the correlation between their estimated CV risk and serum estradiol.

## Materials and methods

This study was a cross-sectional study conducted in the General Outpatient Clinic of a government-owned tertiary hospital in South-Western Nigeria. Ethical clearance for the study was obtained from the institution's ethical review Board. (UI/EC/19/0584). Informed consent was sought and obtained from each study subject recruited. Study population were postmenopausal women aged 46-65years and estimated sample size was 271. Inclusion criteria included consenting postmenopausal women (PMW) within the stated age bracket who had not had their monthly menstruation for at least one-year duration. Exclusion criteria included PMW who needed urgent medical attention or had conditions such as acute psychotic disorder, acute presentation of depression or mania. All PMW who presented at the clinic were purposively identified daily. PMW who met the inclusion criteria were daily recruited consecutively until the estimated sample size was achieved. Study lasted for a period of eight weeks.

An interviewer administered questionnaire was used to collect data relating to the socio-demographic history and medical history (hypertension, diabetes mellitus and dyslipidaemia). Blood pressure and anthropometric measurements such as weight, height, waist and hip circumference were carried out. Height was recorded in metres with a stadiometer manufactured by Seca Corporation, Columbia, Maryland, United States of America. Weight in kilogram (kg) was measured with a standardized weighing scale (Seca model 220). The participants' weight was obtained while standing in light clothing, facing forward without footwear. The hip circumference (HC) was taken at the widest portion of the buttocks while the waist circumference (WC) was measured at a point midway between the iliac crest and the lower costal margin. Measurement was to the nearest centimetres. Abdominal obesity was assessed using the waist-hip ratio. Values greater than 0.85 was classified as abnormal.

Laboratory analysis of fasting lipid profile and serum estradiol level was also done. Six millilitres of whole blood were collected and aliquoted in appropriate sample bottles for analysis of fasting plasma glucose, fasting lipid profile and serum oestradiol (E2) respectively using COBAS 411 blood chemistry analyser.

Specific metabolic CVD risk markers of interest in this study were hypertension, diabetes mellitus and dyslipidaemia. Hypertension was defined as blood pressure of greater than or equal to 140/90mmHg or having been on drug treatment for hypertension. Diabetes mellitus (DM) was defined by a fasting plasma glucose of ≥7.0mmol/L or having been on drug treatment for DM while dyslipidaemia was defined by an abnormal value in any of triglyceride (TG) (> 1.69mmol/l), low density lipoprotein (LDL) (> 2.59mmol/l), high density lipoprotein (HDL) (< 1.29mmol/l) or total cholesterol (TC) (> 5.17mg/dl). Patients that have been on drug treatment for dyslipidaemia were also classified as having a dyslipidaemia^7^. The CV risk profile of respondents was predicted using the AIP (Atherogenic Index of Plasma) and the Framingham CVD risk calculator by R.B. D' Agostino and M.J. Pencina. Data was analysed using SPSS (Statistical Package for Social Sciences) version 23 while data was checked for normality using histogram and stem plots. Descriptive statistics such as means, medians and standard deviations was used to summarize continuous variables- age, height, BMI while frequencies and proportions was used for qualitative data. Association between WHR, estradiol and each cardiovascular risk marker was determined respectively using Chi-square for categorical data and student t-test to compare mean values of continuous variables. Correlation analysis was done to assess the relationship between cardiovascular risk profile and estradiol level. Regression analysis was done to identify factors (including sociodemographic characteristics) that predicted CV risk markers. Level of significance was set at < 5%.

## Results

A total of 271 respondents participated in this study. The mean age was 57.8±5.5 years while the modal age group was 61-65years. Many (43.5%) had secondary education, followed by primary education (21.8%). Majority (81.5%) were married and were mostly Christians (72.0%). Other details of socio-demographic characteristics are as shown in table 1 below.

**Table 1:** Socio-demographic characteristics of respondents (n=271)

Variables	Frequency	Percentage
**Age Group (years)**		
46-50	28	10.4
51-55	67	24.7
56-60	67	24.7
61-65	109	40.2
**Education**		
No formal education	40	14.8
Primary	59	21.8
Secondary	118	43.5
Tertiary	54	19.9
**Marital status**		
Married	221	81.5
Unmarried	50	18.5
**Religion**		
Christianity	195	72.0
Islam	76	28.0
**Occupation**		
Self employed	26	9.6
Civil servant	46	17.0
Retiree	57	21.0
Trading/business	142	52.4
**Ethnicity**		
Yoruba	241	88.9
Igbo	13	4.8
Others	17	6.3

### Lifestyle and clinical characteristics of cespondents

Just 9 (3.3%) respondents used alcohol while none of them smoked tobacco. Diabetes mellitus was present in 48 (17.7%), hypertension in 176 (64.9%), while 253 (93.4%) of the respondents had dyslipidaemia. Details of their mean cholesterol levels and other clinical characteristics is shown in table 2 below.

**Table 2:** Lifestyle and Clinical characteristics of respondents (n=271)

Variables	Mean	Standard Deviation
BMI (kg/m^2^)	28.2	6.3
Total Cholesterol (mmol/l)	4.9	1.0
Low-density Lipoprotein (mmol/l)	3.5	1.0
Triglyceride (mmol/l)	1.2	0.6
High Density Lipoprotein (mmol/l)	0.9	0.3
Waist circumference(cm)	94.6	12.6
Hip circumference(cm)	109.2	11.9
Waist-Hip Ratio	0.87	0.1
Estrogen level*	18.35	7.81

* Median (Interquartile range) used for serum estradiol (did not follow normal distribution)

### Relationship between abdominal fat (Waist-Hip Ratio), serum estradiol and cardiovascular risk markers

There was a statistically significant association between hypertension and WHR (p= 0.01) while diabetes mellitus had no significant association with WHR. Both DM and hypertension had no statistically significant association with serum estradiol level. There was weak positive correlation between each of LDL, TC, TG and WHR while a weak negative correlation existed between HDL and WHR. The correlation between each of WHR, estradiol and components of fasting lipid profile is shown in table 3 below.

**Table 3:** Relationship between abdominal fat (Waist-Hip-Ratio), serum estradiol and metabolic Cardiovascular risk markers

Waist-Hip Ratio		Variable	Serum Estradiol	
R	p-value		R	p-value
0.017	0.775	LDL	-0.093	0.126
0.005	0.931	TC	-0.127	0.037**
0.205	0.001*	TG	-0.23	0.701
-0.244	0.000*	HDL	-0.111	0.069

* Significant at p≤0.05, Pearson correlation

** Significant at p≤0.05, Spearman Rank correlation (non-parametric statistics)

### Estimated cardiovascular risk profile of respondents

**Figure 1:**
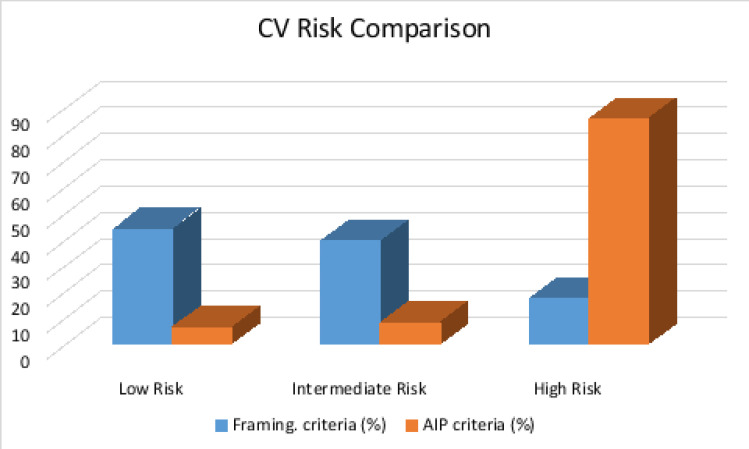
Comparison of Estimated CV Risk using Framingham and AIP criteria

According to the Framingham 10-year CV risk calculator, 43.5%, 39.1% and 17.3% of the respondents had low, intermediate and high risk respectively with a mean risk score of 13.12±7.65 and a range of 1.40 to >30.00. Using the AIP, 85.6%, 8.1% and 6.3% of respondents have high, intermediate and low risk respectively with a mean risk score of 0.47±0.25 and a range of -0.12 to 1.41. Figure 1 is a comparison of the CV risk profile of respondents using the Framingham and AIP criteria while table 4 shows Spearman rank-order correlation between estimated CV risk profile and serum estradiol.

**Table 4:** Correlation between estimated CVD risk profile and serum estradiol

	Framingham criteria Serum Estradiol		Athrogenic Index criteria Serum Estradiol	
Variable	R	P- value	R	P-value
CV Risk	-0.116	0.05*	0.062	0.312

### Regression analysis of factors associated with CV risk markers

Age group was statistically significantly associated with DM (p=0.029) and LDL (p=0.016) at bivariate analysis. Age group, ethnicity and waist-hip ratio remained significantly associated with hypertension following multivariate analysis. Participants who were aged 61-65years were over four times more likely to have hypertension compared to those aged 46-50years (OR= 4.11, C.I. = 1.15-10.95). Also, 10-unit increase in WHR is associated with about two-fold risk of hypertension (OR> 1.73, C.I.= 1.13-2.66). Unmarried (widow, separated) were two times more likely to develop dyslipidaemia and this was statistically significant (OR=2.95, C.I.=1.08-8.08). This is seen in table 5. A unit change in age (1year change in age) is associated with 0.61 increase in TC. Similarly, decrease in E2 level is significantly associated with increase in TC level and vice versa. Each of trader, retiree and self-employed have higher TG level than civil servants when WHR has been controlled for. Similarly, WHR is positively related to TG when occupation has been controlled for. This is shown in table 5.

**Table 5:** Regression analysis of factors associated with CV risk marker

Variables	Adjusted Odds Ratio	p-value	95% Confidence interval	
			Lower	Upper
**Factors associated** **With hypertension**				
Age Group (years)				
46-50	1.00			
51-55	2.95	0.027*	1.13	7.72
56-60	3.26	0.019*	1.21	8.73
61-65	4.11	0.005*	1.54	10.95
**Ethnicity**				
Yoruba	1.00			
Igbo	0.16	0.008*	0.04	0.62
Others	2.24	0.225	0.61	8.26
**Occupation category**				
Civil Servant	1.00			
Trader	1.33	0.460	0.63	2.80
Retiree	2.17	0.128	0.80	5.87
Self-employed	3.02	0.073	0.90	10.12
**Waist-Hip-Ratio_10**				
	1.73	0.012*	1.13	2.66
**Factors associated** **With dyslipidaemia** **Marital Status**				
Married	1.00			
Unmarried	2.95	0.035*	1.08	8.08
**Waist-Hip-Ratio_10**				
	1.32	0.224	0.84	2.07
**Factors associated** **With total cholesterol**				
Age	0.61	-0.28	1.50	0.178
Estradiol Level	-0.05	-0.09	-0.004	0.032*
**Factors associated** **with Triglyceride**				
Occupation cat. (constant)	-18.77			
Trader	10.22	-6.10	26.53	0.219
Retiree	7.75	-11.30	26.80	0.424
Self-employed	37.93	14.30	61.55	0.002
Waist-Hip Ratio_10	13.71	6.36	21.05	0.000*

* Significant at p<0.05

## Discussion

The mean hip circumference (HC) from this study is similar to the cut-off for normal population (97-108cm) while the mean waist circumference (WC) is slightly higher than the normal cut-off for women (78-91cm). Ogwumike et al in the same study location found lower values (waist circumference of 47cm and hip circumference of 43cm). This difference could be because participants in the later study had high level of physical activity and were actively receiving physiotherapy. The average waist-hip ratio (WHR) of respondents was found to be elevated (normal value is <0.80). A higher mean WHR value (0.91) was found by Chhabra et al among PMW in India 8. Sociocultural differences could be responsible for the lower mean WHR level found in the index study.

Among the metabolic cardio markers (TC, LDL, HDL and triglyceride) considered in this study, only HDL and triglyceride had significant correlation with abdominal obesity (Waist-Hip Ratio) in respondents. Similar result was obtained in a study in Thailand among adults aged 18-60 years^9^. This is not surprising as these two types of cholesterol are the ones used in defining metabolic syndrome, which is a constellation of cardiometabolic risk factors^10^. There was a significant association between hypertension and WHR while DM had no significant relationship with WHR. Barroso however, found a significant association between each of hypertension, DM and abdominal obesity while using waist-height ratio as the parameter for abdominal obesity. The difference in finding as it relates to DM in the index study could be due to the different parameter used to evaluate abdominal obesity^11^. The finding from index study, Barroso et al, Lee and Kim in Korean women reveals that combined parameter such as waist-hip ratio or weight-height ratio are better predictors of hypertriglyceridaemia in PMW^11^, ^12^.

A low serum E2 was generally observed among the respondents though a much lower value was found in Enugu 13. The relatively higher serum E2 level found from the index study could be due to the method used for analysis which was electrochemiluminescence, a more sensitive method than ELISA which was the method used in the Enugu study. Studies from India and Dhaka report mean E2 level similar to that obtained from index study using ELISA and radio-immuno assay method respectively^14^. It hence appears that various factors such as race, dietary intake and method of analysis affect serum E2 level in PMW. Total Cholesterol was the only lipid profile parameter found to have statistically significant relationship with serum E2 following logistic regression. This finding is similar to that of Yeasmin et al in Dhaka where a negative correlation was found between each of TC, TG and serum estradiol level, though he found no statistical significance between them^14^. Serum E2 was not found to influence hypertensive status or diabetes mellitus in this study. Similarly, a negative statistically non-significant relationship was found between serum E2 levels and blood pressure levels as well as E2 and blood glucose among PMW in Senegal^15^, ^16^.

Using the framingham risk calculator, close to half of them had a low CV risk profile and less than one-fifth of them had a high-risk profile. This finding is similar to the work by Awotidebe et al.in the same region.^17^. A much lower value for high risk (0.4%) and intermediate risk (9.9%) was obtained in Nepal among PMW in a hospital-based study using the Excel FRS calculator^18^. Regional differences and associated lifestyle differences (Nepalese are mostly vegetarian) can partly explain the higher risk score values obtained in the index study. The mean FRS score from index study is similar to the finding among Cameroonean PMW where CV risk score ranged between 1.2% and >30% with a mean of 13.4 ± 8.7% 19. A comparison of cardiovascular risk score using the FRS and AIP among the respondent showed that more than three quarter of them had high risk using AIP while less than one-fifth of them had high risk using FRS. This finding shows that AIP as a tool in detecting cardiovascular risk is more sensitive than Framingham Risk calculator. This is consistent with findings from other studies where AIP and other novel CV risk markers such as Castelli index have been found to have higher sensitivity^20^. Also, women are known to have a lower short term CV risk as measured by FRS and a higher lifetime CV risk as measured by AIP.^21^

A statistically significant inverse association was found between serum E2 and estimated CV risk score using FRS. This finding supports previous evidence on the cardio-protective nature of E2 in women, with the premenopausal women (with higher E2) having less CV risk compared to the PMW with reduced serum E2 level and increased CV risk^22^. Also, a systematic review among women showed that there is an indirect relationship between exposure to endogenous E2 and risk of cardiovascular events such as stroke^23^. In addition to the known traditional markers of CV risk such as hypertension, DM, physical inactivity, tobacco use, the finding from the index study supports the need for a new CV risk calculator which incorporates factors peculiar to women such as serum E2 level. Other markers of CV risk peculiar to women include history of oral contraceptive use, pregnancy, gestational DM, history of pre/eclampsia or postmeno-pausal E2 use^21^.

Findings from this study as well as previous studies showed that age is a significant predictor of hypertensive status^24^, ^25^. The older the respondents, the higher the odds of being hypertensive. This is partly explained by increased vascular resistance associated with aging. Low educational status, working status of women, socioeconomic status, nuclear family setup and obesity were associated with hypertension among Indian women in a cross-sectional study 26. Ethnicity, as found from this study can significantly influence hypertensive status due to difference in genetic make-up of individuals across various regions of the world. Similarly, age had a significant association with glycaemic status and this is consistent with past finding^27^. These findings support existing evidence that age is a risk factor for onset of chronic non communicable diseases like hypertension and DM. It was found in a Japanese study among PMW that older age and menopause are independent predictors of dysglycaemia^28^.

Total cholesterol and LDL were the specific lipid component found to be influenced by respondents' age. Also, occupation category was also found to influence TG levels. In a study by Agongo et al among Ghanaian PMW in a rural community; socioeconomic status, education, being unmarried and employment status were associated with each of HDL, LDL and TG^29^. Age had positive correlation with each of TC, TG and LDL in PMW in China while a negative correlation was found between age and HDL among them^30^.

## Conclusion

Abdominal obesity and serum E2 significantly influence cardio-metabolic risk markers in women. The AIP has higher sensitivity than FRS in estimation of CV risk among PMW. Lower serum E2 levels is associated with higher estimated CV risk score. In addition to the known traditional markers of CV risk, there is a need for a new CV risk calculator which incorporates factors peculiar to women such as serum E2 level.

## Strength and limitation of study

This study has added to data on serum E2 pattern in African postmenopausal women. Study was conducted in a hospital setting which could make the sample less representative of the entire community. Also, premenopausal women were not studied which made it impossible to assess the direct effect of postmenopausal status on cardiovascular risk in women. Larger community study involving both pre- and postmenopausal women is hereby recommended. Furthermore, studies to evaluate the relationship between novel CV risk markers (such as C-reactive protein, Growth Differentiation Factor, GDF-15 and Brain Natriuretic Peptide) and serum E2 in women is also recommended.

## References

[1] Garcia M, Mulvagh SL, Merz CN, Buring JE, Manson JE (2016). Cardiovascular Disease in Women: Clinical Perspectives. Cm Res.

[2] Piche M-E, Vasan S, Hodson L, Karpe F (2018). Relevance of human fat distribution on lipid and lipoprotein metabolism and cardiovascular disease risk. Current opinion in lipidology.

[3] Chen J-L, Guo J, Mao P, Yang J, Jiang S, He W (2021). Are the factors associated with overweight/general obesity and abdominal obesity different depending on menopausal status?. PLOS ONE.

[4] D'Agostino RB, Pencina MJ, Massaro JM, Coady S (2013). Cardiovascular Disease Risk Assessment: Insights from Framingham. Global heart.

[5] Arnett DK, Blumenthal RS, Albert MA, Buroker AB, Goldberger ZD, Hahn EJ (2019). 2019 ACC/AHA Guideline on the Primary Prevention of Cardiovascular Disease: A Report of the American College of Cardiology/American Heart Association Task Force on Clinical Practice Guidelines. Circulation.

[6] WHO WHO (2020). Noncommunicable Diseases.

[7] Saif-Ali R, Kamaruddin NA, Al-Habori M, Al-Dubai SA, Ngah WZW (2020). Relationship of metabolic syndrome defined by IDF or revised NCEP ATP III with glycemic control among Malaysians with Type 2 Diabetes. Diabetology & Metabolic Syndrome.

[8] Chhabra N, Sodhi K, Kukerja S, Chhabra S, Chhabra S, Ramessur K (2014). High waist circumference-A potential risk factor for premature metabolic syndrome in women irrespective of menopausal status. Integr Mol Med.

[9] Sukkriang N, Chanprasertpinyo W, Wattanapisit A, Punsawad C, Thamrongrat N, Sangpoom S (2021). Correlation of body visceral fat rating with serum lipid profile and fasting blood sugar in obese adults using a noninvasive machine. Heliyon.

[10] Gaillard TR (2018). The Metabolic Syndrome and Its Components in African-American Women: Emerging Trends and Implications. Frontiers in Endocrinology.

[11] Barroso TA, Marins LB, Alves R, Gonçalves ACS, Barroso SG, Rocha GdS (2017). Association of Central Obesity with The Incidence of Cardiovascular Diseases and Risk Factors. International Journal of Cardiovascular Sciences.

[12] Ju Lee B, Yeol Kim J (2015). Indicators of hypertriglyceridemia from anthropometric measures based on data mining. Computers in Biology and Medicine.

[13] Ewenighi C, D U, A B, O J, O L, O G (2014). Reproductive Hormonal Profile of Post-menopausal Women in Ebonyi State, Nigeria. British Journal of Medicine & Medical Research Sciencedomain international.

[14] Yeasmin N, Akther Q, Mahmuda S, Nahar S, Rabbani R, Hasan M (2017). Effect of Estrogen on Serum Total Cholesterol and Triglyceride Levels in Postmenopausal Women. J Dhaka Med Coll.

[15] Yeasmin N, Akhter QS, Mahmuda S, Banu N, Yeasmin S, Akhter S (2017). Association of Hypertension with Serum Estrogen Level in Postmenopausal Women. Mymensingh medicaljournal: MMJ.

[16] Yeasmin N, Akhter Q, Mahmuda S, Hasan M, Rabbani R, Afroz R (2017). Correlation of Estrogen with Serum Insulin and Blood Glucose Levels in Post-menopausal Women. Bangladesh Medical Journal.

[17] Awotidebe T, Adedoyin R, Olola I, Adeyeye V, Akinola O, Mbada C (2014). Cardiovascular Risk Profile of Post-Menopausal Women in a Semi-Urban Community in Nigeria. British Journal of Medicine & Medical Research SCIENCEDOMAIN international.

[18] Bajracharya S, Rai B, Giri R, Joshi R (2018). Risk Stratification of Coronary Heart Disease in Postmenopausal Women Using Framingham Scale in Eastern Nepal. Medical Journal of Shree Birendra Hospital.

[19] Ama Moor VJ, Nansseu JR, Nouaga ME, Noubiap JJ, Nguetsa GD, Tchanana G (2016). Assessment of the 10-year risk of cardiovascular events among a group of Sub-Saharan African post-menopausal women. Cardiology journal.

[20] Adedokun K, Olisekodiaka J, Adetunji A, Muhibi M, Ojokuku H, Akinlawon A (2017). Castelli Risk Index, Atherogenic Index of Plasma, and Atherogenic Coefficient: Emerging Risk Predictors of Cardiovascular Disease in HIV-Treated Patients. Saudi Pharmaceutical Journal.

[21] Wenger N (2017). Tailoring cardiovascular risk assessment and prevention for women: One size does not fit all. Glob CardiolSci Pract.

[22] Iorga A, Cunningham CM, Moazeni S, Ruffenach G, Umar S, Eghbali M (2017). The protective role of estrogen and estrogen receptors in cardiovascular disease and the controversial use of estrogen therapy. Biology of Sex Differences.

[23] Mishra S, Chung H-F, Waller M, Mishra G (2021). Duration of estrogen exposure during reproductive years, age at menarche and age at menopause, and risk of cardiovascular disease events, all-cause and cardiovascular mortality: a systematic review and meta-analysis. BJOG: An International Journal of Obstetrics & Gynaecology.

[24] Ajayi I, Sowemimo I, Akpa O, Ossai N (2016). Prevalence of hypertension and associated factors among residents of Ibadan-North Local Government Area of Nigeria. Nigerian Journal of Cardiology.

[25] Bosu WK, Aheto JMK, Zucchelli E, Reilly ST (2019). Determinants of systemic hypertension in older adults in Africa: a systematic review. BMC Cardiovascular Disorders.

[26] Dhall M, Tyagi R, Kapoor S (2015). Bio-Social Predictors of Hypertension Among Premenopausal and Postmenopausal Women. SAGE Open.

[27] Mohamed SF, Mwangi M, Mutua MK, Kibachio J, Hussein A, Ndegwa Z (2018). Prevalence and factors associated with pre-diabetes and diabetes mellitus in Kenya: results from a national survey. BMC Public Health.

[28] Heianza Y, Arase Y, Kodama S, Hsieh SD, Tsuji H, Saito K (2013). Effect of postmenopausal status and age at menopause on type 2 diabetes and prediabetes in Japanese individuals: Toranomon Hospital Health Management Center Study 17 (TOPICS 17). Diabetes Care.

[29] Agongo G, Nonterah EA, Debpuur C, Amenga-Etego L, Ali S, Oduro A (2018). The burden of dyslipidaemia and factors associated with lipid levels among adults in rural northern Ghana: An AWI-Gen sub-study. PLOS ONE.

[30] Feng L, Nian S, Tong Z, Zhu Y, Li Y, Zhang C (2020). Age-related trends in lipid levels: a large-scale cross-sectional study of the general Chinese population. BMJ Open.

